# AOJNMB Appreciates the Best Contributors in Years 2015-2013

**DOI:** 10.7508/aojnmb.2016.04.001

**Published:** 2016

**Authors:** S. Rasoul Zakavi

**Affiliations:** Editor in Chief, AOJNMB

AOJNMB is striving for excellence. Our journal is publishing its 4^th^ volume and we are delighted to observe on time publication of this journal with important scientific articles.

**Figure 1:**
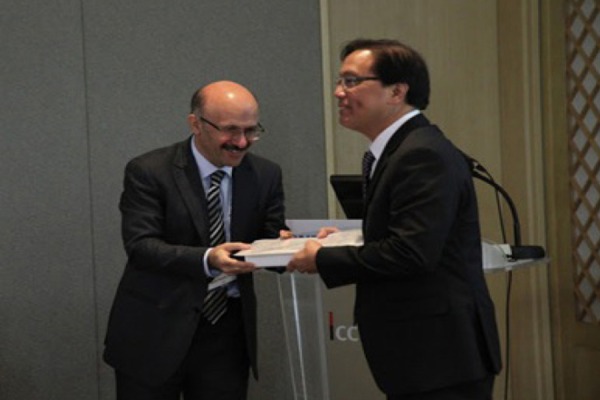
Prof. S. Rasoul Zakavi (left) gives the AOJNMB “Best Reviewer Award” to Prof. Jerry Obaldo (Right) in JICC, Jeju Korea

On November 2015, 11^th^ Asia Oceania Congress of Nuclear Medicine & Biology (AOCNMB) was held in Jeju International Convention Center (JICC) in Korea with hundreds of participants and the abstracts of the meeting were published as a supplement issue of the AOJNMB (1). The 11^th^ AOCNMB meeting was a great opportunity for me to thank the best contributors of the AOJNMB in the last three years. Actually, AOJNMB awarded three contributors for their invaluable effort in years 2013-2015. Prof. Seigo Kinuya was awarded as our “Best Associate Editor” for the highest number of successful editorship, Prof. Henry Bom as “Top Contributor” with the highest number of reviewed articles and Prof. Jerry Obaldo as the “Best Reviewer” for his rapid, critical and instructive reviews. I handed over theses awards to the recipients, in national delegate assembly session of AOFNMB on 3^rd^ of November 2015 in JICC (Figure 1). This important event was also a good opportunity to introduce AOJNMB to the researchers and scientists. AOJNMB had a booth in exhibition hall of JICC and we distributed hundreds of printed journals from previous issues of AOJNMB among visitors. Also I talked about the “current activities and future plans” of AOJNMB in ARCCNM session on 3^rd^ of November 2015 in JICC.

In year 2015, we tried to harmonize the process of article review in AOJNMB, by providing a guide for reviewers and requesting them to evaluate articles in a systematic process. The standardized review of the articles could significantly improve quality of published articles and prevent incomplete evaluations. We invite physicians and scientists to join AOJNMB as a reviewer. An application form is available on the website of AOJNMB for this purpose. We continue working on this subject to further improve the review process and ensure rapid, informative and instructive review of the manuscripts.

Also we are planning workshops on scientific writing, statistical analysis, methodology and article review for empowerment of the nuclear medicine physicians and scientists in the region. Additionally, we plan to refresh our editorial board by inviting active enthusiastic scientists to join us in the editorial team.

AOJNMB is becoming more popular in the Asia Oceania region and among nuclear medicine physicians and scientists. That is reflected by the increasing number of submitted manuscripts to our journal. However we are still far away from proper contribution of great number of researchers in China, Korea and India (2). We should further advance introduction of AOJNMB in these countries. The editorial board members from these countries could play a big role in this regard. Anyhow we need a commitment from all editorial board members, to submit interesting and important original articles to AOJNMB and to introduce AOJNMB in every lecture in every national and international meeting, congresse and seminar. We also request every author or editorial member of AOJNMB to encourage his/her colleagues to visit the AOJNMB website (http://aojnmb.mums.ac.ir) and to submit articles to the journal. To introduce this journal to other colleagues, it is a good idea to download the full text of an article from AOJNMB website and send it to a colleague, if you think it fits with the research scope of your colleague. Also this may improve citation of our journal that is one of the main issues for indexing of AOJNMB in the ISI web of knowledge. We need to get citation for articles of AOJNMB, in the articles published in the ISI journals.

Being indexed in the major databases of medical literature (i.e: Pubmed,Scopus, ISI,..) is an important factor for visibility of the journals. In the last 2 years, we applied for indexing in many important indexing databases and the good news is that AOJNMB has passed successfully the first step of evaluation for indexing in PubMed Central and is waiting for the final technical evaluation. However some other indexing databases, like Scopus have a long backlog of journals for evaluation and it takes more than a year for being evaluated in these databases! Anyhow we applied for inclusion in most of the important indexing databases and wish to be indexed in all of important databases by the end of 2017. Also in year 2015 we obtained digital Object Identifier (DOI) for AOJNMB articles. This again will improve the visibility and electronic access to the articles of AOJNMB.

Finally, it is a great pleasure and honor for me and the team in AOJNMB office, to use this opportunity to congratulate you for the New Year 2016 and wish you health, peace and prosperity.
